# Assessment of Arabic Web-Based Knowledge About Clear Aligners: An Infodemiologic Study

**DOI:** 10.7759/cureus.46879

**Published:** 2023-10-11

**Authors:** Mahmoud Alsulaimani, Muath S Alassaf, Hatem H Hamadallah, Aseel M Aloufi, Khalid N Alturki, Ahmed M Almghamsi, Rawah T Eshky

**Affiliations:** 1 Orthodontics and Dentofacial Orthopedics, Taibah University, Madina, SAU; 2 Dentistry, Taibah University, Madina, SAU; 3 Dental Education, Taibah University, Madina, SAU; 4 Oral and Maxillofacial Surgery, King Fahad General Hospital, Madina, SAU

**Keywords:** online health information seeking, patient-centered information, orthodontics, readability, web-based knowledge, clear aligners, discern

## Abstract

Background: Orthodontic treatments aim to enhance dental aesthetics, functionality, and long-term oral health. Clear aligners have gained popularity as an aesthetic and convenient option for patients seeking orthodontic correction. However, the quality and readability of online Arabic patient-centered information regarding clear aligners has not been studied yet. The aim of our study is to investigate the quality and readability of Arabic patient-centered information about clear aligners.

Methods: We conducted an extensive evaluation of Arabic web-based content pertaining to clear aligners using three prominent search engines. Eligible websites were categorized based on specialization, organizational affiliation, material type, and presentation style. We assessed website quality and reliability using the DISCERN instrument, Journal of American Medical Association (JAMA) benchmarks, and Health on the Net (HON) code. In addition, we measured readability using the Flesch Reading Ease Score (FRES), Simplified Measure of Gobbledygook (SMOG), and Flesch-Kincaid Grade Level (FKGL).

Results: Out of 600 search results, 195 websites met the inclusion criteria. None of the websites were HON-code accredited. DISCERN assessments revealed low content quality, with none of the websites achieving high-quality status. The JAMA benchmarks showed limited compliance with the four items, with currency being the most frequently achieved. Readability assessments indicated generally high readability, with FKGL scores suggesting easy comprehension for the average readers.

Conclusion: While Arabic web-based information on clear aligners is highly readable, its credibility and quality require significant improvement. Websites should adhere to medical information standards, subject content to rigorous assessments, and seek accreditation to ensure reliability. Enhancing the accessibility and comprehensibility of health-related content will empower individuals to make informed health decisions. Addressing limitations, such as social media and video content evaluation, and conducting comparisons with English websites in future research will provide a more comprehensive understanding of the landscape of online orthodontic information.

## Introduction

Orthodontic treatments have long been used to correct dental misalignments and improve the aesthetics and functionality of smiles [1]. Orthodontic treatments aim to create an aesthetic smile with proper occlusion for improved chewing function and long-term dental health. Common types of malocclusions that can be treated with orthodontics include crowding, spacing, overjet, deep bite, open bite, and skeletal discrepancies in jaw size or position [1]. Orthodontic treatments have the benefits of improved self-esteem, correction of speech issues, easier oral hygiene, relief from jaw joint problems, and long-term dental health with properly aligned teeth [2]. There are various types of fixed and removable orthodontic appliances that are used to gradually move teeth into their ideal position [3]. Fixed appliances, such as traditional metal braces, or removable appliances, such as clear aligners and retainers, are used to maintain teeth in corrected positions. Headgear, elastics, or functional appliances may also be used along with braces for certain malocclusions [3]. Clear aligners have become a popular alternative to traditional braces for orthodontic treatments in recent years, as they are invisible when worn, providing an aesthetic advantage that appeals to many patients. In addition, clear aligners can be conveniently removed for eating, oral hygiene, and flossing. This removability makes maintaining good oral health and diet much easier during treatment compared to braces [4]. It has been reported that these removable appliances are easy to use, comfortable, and safe and have a simple design and function [5]. Because of these features, removable appliances are well-suited for early orthodontic treatments during the mixed dentition stage. Generally, patients are satisfied with their orthodontic clear aligners. According to studies, patients are satisfied with the aesthetics, comfort, and convenience of the use of clear aligners. When compared to conventional braces, clear aligners demonstrate higher levels of satisfaction with treatment outcomes [6].

As the popularity of clear aligner systems continues to increase, there is still some uncertainties about their effectiveness [7]. While clear aligners are being used for more and more complex cases, they still cannot treat all types of malocclusions [8]. Clear aligners are effective in treating mild to moderate crowding or spacing, posterior expansion, lower incisor extraction cases, and distal tipping of molars, intrusion of one or two teeth; however, they are not as equally effective in more difficult movements, such as extrusion, correction of severe rotations, molar uprighting, and closure of extraction spaces, which are known to be more difficult to achieve with aligners [7]. Currently, the use of temporary anchoring devices in conjunction with clear aligners has expanded the spectrum of treatments feasible with aligners even more, particularly for difficult movements [9].

Orthodontists should prioritize time efficiency since it not only satisfies patients and allows them to complete treatment faster but also allows the orthodontist to help more patients by spending less time with each one in the clinic [10]. A systematic review concluded that both aligners and braces can effectively improve malocclusion. However, fixed braces were found to be more effective in achieving sufficient occlusal contacts, improving transverse width, and controlling posterior buccolingual inclination. Clear aligners demonstrated good control in maintaining teeth inclination and shorter treatment durations in no extraction cases. Therefore, orthodontists should consider the characteristics of these two orthodontic appliances when making treatment decisions based on the specific needs of each case [11].

In today's digital era, the Internet has emerged as a powerful hub of health knowledge, wielding significant influence over patients' decision-making processes [12]. With a few clicks, individuals can access a vast array of health information, shaping their interactions and overall satisfaction with healthcare providers [12]. Patients frequently use the Internet to access health information, which impacts their interactions and satisfaction with healthcare providers. Patients may develop misconceptions from poor-quality online information [12]. An increasing amount of web-based content covers clear aligners of orthodontic therapy, shaping public awareness and utilization of aligners [13]. In addition, manufacturer websites clarify aligner treatment techniques and advertise their brands, while social media share patient experiences. Video sites like YouTube host demonstrations of aligner placement and reviews [14]. However, this approach may have a positive or negative impact on the treatment process, depending on the quality and reliability of the information obtained [15]. Many studies have been conducted to assess web-based information regarding orthodontic clear aligners [14,16,17] and have shown low- to moderate-quality web-based information. Furthermore, as Internet access grows in regions, such as the Middle East and North Africa, Arabic speakers are increasingly relying on online health information [18]. However, the quality of Arabic language health information online can be problematic due to inaccurate translations, cultural misunderstandings, and lack of accreditation [17]. With around 422 million Arabic speakers, it is the fifth most spoken language globally [19]. It is crucial that health information in Arabic be precise and complete. Although many health topics have been thoroughly evaluated for their web-based content, there is still a considerable lack of assessment regarding web information on clear aligners in Arabic.

Assessing the quality and accuracy of online Arabic clear aligner information is vital given the millions of access health information in the Internet. More studies focused on evaluating Arabic language orthodontic aligner websites, social media, and other online resources would help address this study gap. This would ensure that Arabic-speaking patients have access to reliable and comprehensive sources when researching clear aligner treatment options online. To the best of our knowledge, there are no studies that have evaluated the quality and readability of Arabic health websites about clear aligners. Thus, the aim of our study is to investigate the quality and readability of Arabic patient-centered information about clear aligners. This study seeks to provide a benchmark for evidence-based information and guidance for Arabic-speaking individuals and professionals.

## Materials and methods

Website research and selection

The Arabic terms for "clear aligner" and “Invisalign” have been entered into three search engines: Google, Bing, and Yahoo. Eligibility criteria were set to exclude websites with non-Arabic content, duplicate websites, websites with brief mentions of clear aligners, scientific articles, recordings, social media content, promoted links, advertisement banners and pop-ups in the website, news articles, unrelated content, and websites necessitating log-in/payment.

Then, the included websites were categorized in accordance with Ni Riordain and McCreary's classification [20]. Based on specialization (partially or exclusively related to clear aligners), level of organization (commercial, a nonprofit organization, university/medical center, or governmental), type of materials (medical facts, clinical trials, question and answers, and personal stories), and presenting style (images, recordings, and videos).

Quality assessment

The quality and reliability of the websites were evaluated using three different markers: the Journal of the American Medical Association (JAMA) benchmarks for website analysis [21], Health on the Net (HON) evaluation [22], and DISCERN evaluation instrument [23].

The American Medical Association created the JAMA markers in 1997. It is based on four elements: disclosure (openly shows ownership and donations, sources of funding, and conflicts of interest), currency (using information about the dates of content sharing, improvements, and editing), authorship (recognizes researchers and contributors), and attribution (clearly stating the sources of information).

A standardized tool called the DISCERN instrument was used to rate the accuracy of health information. It includes three categories and 16 items with points varying from 1 to 5. Points range from complete approval (5) to full rejection (1). Items 1 through 8 and 9 through 15 in the first two components concern validity, and the last item asks for a general assessment. Following that, the whole points are further divided into low (16-32), moderate (33-64), and high (65) quality.

Furthermore, a widely recognized credibility mark for accurate online health-related information is the HON code. HON code accreditation is available for health-related websites, which is assessed by applying eight standards for excellent quality and openly disclosed data. If a website meets these standards, it obtains an HON seal for a period of one year, with annual evaluations required in order to maintain it.

Readability evaluation

The readability of Arabic text was assessed using three different measures: the Flesch Reading Ease Score (FRES), Simplified Measure of Gobbledygook (SMOG), and Flesch-Kincaid Grade Level (FKGL) scores. The SMOG score increases for texts that are harder to comprehend. The FKGL score also measures the difficulty of a text on a scale from 0 to 18, with higher scores indicating more challenging readability. Meanwhile, the FRES ranges from 0 to 100, with higher scores indicating easier readability. Scores below seven were decided to be satisfactory for FKGL and SMOG in terms of readability; however, a score of 80 or more was accepted as adequate for FRES [24-26].

Ethical considerations

As this study utilized information that is publicly available with no human-related or identifying contents, ethical approval and consent form was not required.

Data analysis plan

The statistical data were analyzed using the IBM SPSS Statistics for Windows, version 25 (released 2017; IBM Corp., Armonk, New York, United States). According to the Kolmogorov-Smirnov test, the normally distributed data were presented as mean with standard deviation (SD), while non-normally distributed data were reported as median with interquartile range (IQR). For comparative tests, a P-value of 0.05 or less was considered statistically significant.

## Results

Included websites and categorization

**Figure 1:**
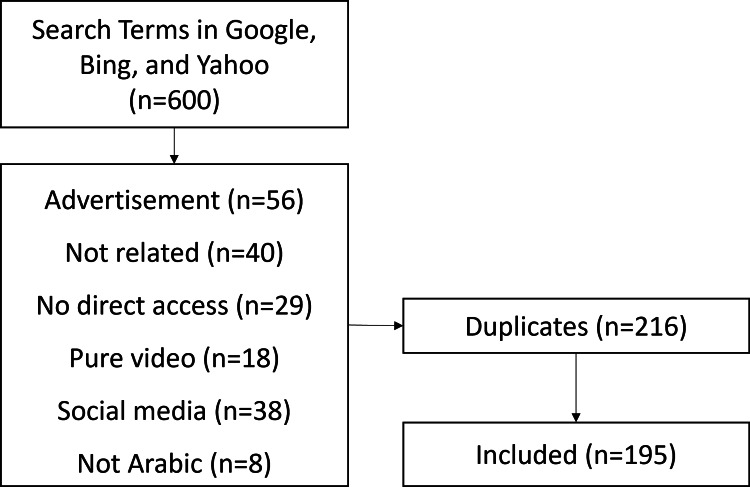
Flow chart of the search strategy

The number of checked search results was 600, the first hundred websites per search engine per term. Among them, 195 websites were eligible for assessment. The most common reason for website exclusion was advertisements on 56 websites. Of the 411 websites, 216 were duplicated. Figure 1 shows a summary of the search strategy and reasons for website exclusion.

According to the website affiliation, websites belonging to universities or medical institutions were most common and accounted for 101 (51.8%) websites, followed by commercial websites with 84 (43.1%) and only 10 websites belonging to non-profit organizations. None of the websites belonged to the government. Most of the websites were partially related to orthodontic treatments with clear aligners, while only 13 (6.7%) websites were exclusively related to clear aligners. The content type was mainly medical facts in 181 (92.8%) websites, and 70 (35.9%) websites contained questions and answers. The content was presented with images on 165 (84.6%) websites, while none of the included websites had audio in addition to text in the data presentation. Table 1 summarizes the categories of the websites according to affiliation, specialization, content type, and presentation.

**Table 1:** Affiliation and content type and presentation of the included websites (n=195) Data presented as frequency (n) and percentages (%)

Category	Criteria	n (%)
Affiliation	Commercial	84 (43.1%)
	Non-profit organization	10 (5.1%)
	University/medical center	101 (51.8%)
	Governmental	0
Specialization	Exclusively related	13 (6.7%)
	Partly related	182 (93.3%)
Content type	Medical facts	181 (92.8%)
	Clinical trials	5 (2.6%)
	Human interest stories	25 (12.8%)
	Question and answer	70 (35.9%)
Content presentation	Image	165 (84.6%)
	Video	18 (9.2%)
	Audio	0

Quality assessment

The quality of websites was evaluated by three tools: the DISCERN instrument, JAMA benchmarks, and HON code. For the HON code, none of the websites was sealed with the HON code.

The DISCERN assessment revealed a median total score (sum of questions 1 to 15) of 31 (10.5), a reliability score (questions 1 to 8) with a median of 16 (5.5), and, lastly, a treatment score (questions 9 to 15) median of 14.5 (5). The median of each question with the maximum and minimum scores is reported in Table 2. Questions four, 12, and 13 had the lowest median scores, while the highest score was reported in the third and sixth questions. The median and IQR of the total, reliability, and treatment scores of the websites according to their affiliation are summarized in Table 3.

**Table 2:** DISCERN evaluation for the included websites (n=195) Data presented as median with interquartile range (IQR), maximum (Max), and minimum (Min).

Domain	DISCERN question	Median (IQR)	Max	Min
Reliability	Q1. Explicit aims	3 (1)	5	1
	Q2. Aims achieved	2.5 (1)	5	1
	Q3. Relevance	3 (1.5)	5	1
	Q4. Explicit sources	1 (0)	5	1
	Q5. Explicit date	1.5 (2.5)	5	1
	Q6. Balanced and unbiased	3 (1.5)	5	1
	Q7. Additional sources	1.5 (1)	4	1
	Q8. Areas of uncertainty	1.5 (1)	4	1
Treatment options	Q9. How treatment works	2 (2)	5	1
	Q10. Benefits of treatment	2.5 (1.5)	5	1
	Q11. Risk of treatment	2 (1.5)	5	1
	Q12. Effects of no treatment	1 (0.5)	3	1
	Q13. Effects on quality of life	1 (0.5)	3	1
	Q14. All alternatives described	1.5 (1)	4.5	1
	Q15. Shared decision	2 (2)	5	1
Overall rating	Q16. Overall quality rating	2.5 (1)	4	1

**Table 3:** Quality and readability of the included websites based on their affiliation reported as frequency and percentage (n =195) Data presented as frequency with interquartile range (IQR). NA, Not Applicable; * Significant at level 0.05 or less; JAMA: Journal of American Medical Association; FRES: Flesch Reading Ease Score; FKGL: Flesch-Kincaid Grade Level

Variable	Variable type	Commercial	Non-profit organization	University/medical center	Total	P-value
Number of achieved JAMA items per website	None	26 (13.3%)	1 (0.5%)	49 (25.1%)	76 (39.0%)	0.000*
	One	22 (11.3%)	2 (1.0%)	23 (11.8%)	47 (24.1%)	
	Two	36 (18.5%)	4 (2.1%)	26 (13.3%)	66 (33.8%)	
	Three	0 (0.0%)	3 (1.5%)	3 (1.5%)	6 (3.1%)	
JAMA items	Authorship	42 (21.5%)	8 (4.1%)	37 (19.0%)	87 (44.6%)	0.013*
	Attribution	1 (0.5%)	3 (1.5%)	4 (2.1%)	8 (4.1%)	0.000*
	Currency	51 (26.2%)	8 (4.1%)	43 (22.1%)	102 (52.3%)	0.010*
	Disclosure	0 (0.0%)	0 (0.0%)	0 (0.0%)	0 (0.0%)	NA
DISCERN	Low	47 (24.1%)	3 (1.5%)	66 (33.8%)	116 (59.5%)	0.064
	Medium	37 (19.0%)	7 (3.6%)	35 (17.9%)	79 (40.5%)	
	High	0 (0.0%)	0 (0.0%)	0 (0.0%)	0 (0.0%)	
FRES	Readable	77 (39.5%)	8 (4.1%)	95 (48.7%)	180 (92.3%)	0.270
	Difficult	7 (3.6%)	2 (1.0%)	6 (3.1%)	15 (7.7%)	
FKGL	Readable	59 (30.3%)	6 (3.1%)	66 (33.8%)	131 (67.2%)	0.689
	Difficult	25 (12.8%)	4 (2.1%)	35 (18.0%)	64 (32.8%)	

The JAMA benchmarks had four items: authorship, attribution, disclosure, and currency. The currency was the most commonly achieved in 102 (52.3%) websites, followed by authorship in 87 (44.6%) websites. Attribution was the least reported in 8 (4.1%) websites. The disclosure was not reported on any of the websites. The distribution of the achieved JAMA items according to website affiliation showed statistically significant differences in authorship, attribution, and currency, as shown in Table 3. For the number of achieved JAMA items per website, none of the websites achieved the four items in the same websites; in addition, none of the items achieved in 76 (39%) websites. The number of achieved items per website is shown in Table 3. The distribution of the number of achieved items per website according to websites’ affiliation showed statistically significant differences (p-value <0.001).

Readability assessment

The FRES of 100 is the maximum and 37.7 is the minimum, with a median of 99.8 (13.3). FRESs did not show any statistical differences based on website affiliation. For the FKGL test, the maximum and minimum scores were 9.9 and 0.04, respectively. The median score for FKGL was 5.1 (5.3), with no statistical differences among different affiliations. The SMOG test showed that all websites are readable with a median of 3.99 (0.013) and maximum and minimum scores of 4 and 2.9, respectively. A statistical difference was found across different affiliations (p-value=0.011); more details are shown in Table 4.

**Table 4:** Comparison between medians with interquartile range according to websites’ affiliation (n=195) Data presented as frequency (n) and percentages (%). * Significant at a level of 0.019 between university/medical center and commercial, also significant at a level of 0.004 between university/medical center and non-profit organization. ** Significant at a level of 0.022 between university/medical center and commercial, also significant at a level of 0.019 between university/medical center and non-profit organization. FRES: Flesch Reading Ease Score; FKGL: Flesch-Kincaid Grade Level; SMOG: Simplified Measure of Gobbledygook

Variable	Commercial	Non-profit organization	University/medical center	P-value
Overall	30.8 (11)	35.8 (11.4)	30.5 (9.5)	0.353
Reliability	16.8 (6)	21.8 (8.4)	15 (5.8)	0.003*
Treatment	14 (5.5)	13 (1.8)	15 (5.8)	0.080
FRES	98.7 (12.3)	98.1 (27.3)	100.1 (13.9)	0.811
FKGL	5.4 (4.5)	5.6 (7.7)	5 (6)	0.998
SMOG	3.997 (0.007)	3.999 (0.004)	3.995 (0.016)	0.011**
Words	710 (493)	925.5 (1016)	761 (665)	0.658
Sentences	28.5 (27)	20.5 (61)	31 (26)0	0.597

The maximum count of words on a website was 2847, while the minimum was 26, with a median of 721 (609). In accordance, the sentence count had a maximum of 152 and a minimum of eight sentences, with a median of 30 (26). In comparison based on affiliations, no significance was found in the word or sentence count.

## Discussion

The primary objective of orthodontic treatments is to achieve a harmonious interplay between an aesthetically pleasing smile and proper occlusal alignment, thus enhancing masticatory function and promoting optimal oral health in the long term. Malocclusion is a prevalent condition that results in the loss of proper masticatory function and/or unattractive smile [5]. In the era of the Internet, it is not surprising that patients use online sources to knowledge themselves about their conditions and treatment options. In the field of orthodontic treatments, multiple studies were conducted to evaluate the quality of patient-centered online content in terms of YouTube videos [27], social media tweets [28], and different website contents [29]. The aim of this study is to evaluate the quality and readability of Arabic online patient-centered information about orthodontic clear aligners in the most commonly used search engines.

A similar study by Alpaydın et al., which evaluated the English content about clear aligners, included 111 websites out of 150 websites; although this study included double this number, it excluded about 400 websites. The same exclusion criteria were considered in both studies, with most of the exclusion in this study being due to the presence of advertisement banners and pop-ups [29].

In the Arabic content, of this study, none of the websites had the HON seal compared to six websites in Alpaydın et al.’s study [29]. The HON code seal on a medical website indicates understandable, accessible, and reliable information but does not guarantee the accuracy of the presented content [22].

In the DISCERN evaluation, the total, reliability, and treatment scores were close to the English content 30, 15, and 14.5, respectively. This shows that the quality of the content is similar in both languages and indicates low-quality content [29]. A deeper look at the numbers shows that in both languages, the non-profit organizations had the highest scores, indicating moderate quality, and the lowest number of websites. About 60% of the websites in this study had low content quality, and the rest were moderate. Badly, none of the websites scored higher than 65, so none had high-quality content. Most of the pitfalls were in questions four, 12, and 13. The query posed in the fourth question pertains to the presence of references that can serve as a support system for the content presented. It is widely acknowledged that credible and authentic information necessitates the backing of appropriate sources. The questions about the effect of no treatment (12) and the effect on the quality of life (13) also recorded poor scores. Question 13 had the lowest score in the English study too [29]. The non-profit organization websites have demonstrated remarkable DISCERN scores, as evidenced by their superior performance in almost all sections. Specifically, the median total DISCERN score of these websites was 35.8, which was significantly higher than the other groups, as listed in Table 4. DISCERN is an easy-to-understand and comprehensive reliable tool to quickly assess the reliability and quality of online health-related topics; it is advantageous that physicians know it and educate their patients about it to carefully obtain their knowledge, especially in long-term treatments, such as orthodontic treatments [23].

JAMA benchmarks were set by JAMA to evaluate four items: authorship, attribution, disclosure, and currency. In this study, the four items were not achieved in a single website, in contrast to the English Alpaydın et al.’s study, in which six websites achieved all the items [29]. Currency was the top-ranked item, and none had achieved disclosure. This represents a complete contrary, in which, in English contents, disclosure was the top-ranked item [29]. In more than one web-based content evaluation in the field of dentistry, disclosure was also the lowest achieved item [29,30]. The number of achieved JAMA items per website was relatively high for non-profit organizations (Table 3); this goes in line with the findings of a study about the web-based knowledge of implant bone graft [30]. Universities or medical centers belonged to websites that accounted for a quarter of the websites that did not achieve any JAMA item; this was also true in a similar study [30]. Although the JAMA evaluation is not as detailed as DISCERN, it can give an overview of the quality of the website in a more rapid way.

The readability of patient-centered knowledge is of huge importance, as it is not reasonable to provide accurate and reliable content that cannot be understood by the reader. The use of medical and complicated words and explanations makes it difficult for the patient to understand the information from the physician, not to mention reading it from an online source [31]. The SMOG readability test showed that all websites had acceptable readability (scored more than seven); however, the SMOG test depends in calculation on the presence of polysyllabic words, which is not common in Arabic; this can explain the result of readable text in SMOG but not in FRES or FKGL tests [24].

The majority of the websites included in the analysis displayed a lower FKGL score, signifying that they were easily comprehensible for the average Internet user. This conclusion was further substantiated by the SMOG results, confirming that all of the aforementioned websites could be understood with ease by an individual with a middle school level of education. In addition, the high FRE score indicates that the websites were indeed simple to understand. These findings are consistent with other Arabic content evaluation studies [24,32]. Compared to English content, Arabic content is more easily readable [29,30].

The comprehensibility of Arabic online patient-focused information pertaining to clear aligners is notable. However, its credibility leaves much to be desired. As such, there is a pressing need to enhance the quality of this information. Websites should adhere to established medical information standards and subject their content to various assessment tools in order to ensure its veracity. This initiative seeks to enhance the accessibility and comprehensibility of health-related content, thereby empowering individuals to make informed decisions about their health. By sharing this study with relevant stakeholders, we hope to foster a culture of health education and awareness and ultimately improve health content for the Arabic-speaking population.

Limitations and recommendations

It is important to acknowledge certain limitations in our study. First, we did not take into consideration social media tweets and YouTube videos. Second, the readability tests employed were not specifically formulated for the assessment of Arabic text, although they may still be applicable. It is imperative for future research to address these limitations in a more comprehensive manner. Finally, we suggest that assessors evaluate both Arabic and English websites in order to obtain a more precise comparison.

## Conclusions

While Arabic web-based information on clear aligners is highly readable, its credibility and quality require significant improvement. Websites should adhere to medical information standards, subject content to rigorous assessments, and seek accreditation to ensure reliability. Enhancing the accessibility and comprehensibility of health-related content will empower individuals to make informed health decisions. Addressing limitations, such as social media and video content evaluation, and conducting comparisons with English websites in future research will provide a more comprehensive understanding of the landscape of online orthodontic information.
